# P-1693. Group A Streptococcus at Wadsworth Center; Surveillance for Antimicrobial Resistance

**DOI:** 10.1093/ofid/ofaf695.1867

**Published:** 2026-01-11

**Authors:** Kate T Wahl, Erin Klingbeil, Andrew Peifer, Anna Kidney, Elizabeth Owuor Bielli, Linnell Randall, Patrick Boynton, Evan Owens, Jill Hayes, Lori Tyler, Lynn Leach, John Jurczynski, Catharine Prussing, Kimberlee A Musser, Kara Mitchell

**Affiliations:** NYSDOH Wadsworth Center, Albany, NY; NYSDOH Wadsworth Center, Albany, NY; NYSDOH Wadsworth Center, Albany, NY; NYSDOH Wadsworth Center, Albany, NY; NYSDOH Wadsworth Center, Albany, NY; NYSDOH Wadsworth Center, Albany, NY; NYSDOH Wadsworth Center, Albany, NY; NYSDOH Wadsworth Center, Albany, NY; NYSDOH Wadsworth Center, Albany, NY; NYSDOH Wadsworth Center, Albany, NY; NYSDOH Wadsworth Center, Albany, NY; NYSDOH Wadsworth Center, Albany, NY; NYSDOH Wadsworth Center, Albany, NY; Wadsworth Center, New York State Department of Health, Albany, NY; NY Wadsworth Lab, Albany, New York

## Abstract

**Background:**

Group A *Streptococcus* (GAS) is highly contagious and can cause invasive infections including bacteremia, sepsis, and necrotizing fasciitis. Thus, it is important to limit transmission and treat infections promptly and appropriately. In 2019, the Centers for Disease Control and Prevention (CDC) listed resistant GAS as a concerning threat due to increasing rates of clindamycin (CLI) and erythromycin (ERY) resistance. The Wadsworth Center (WC) is one of 10 laboratories in the country funded to perform GAS surveillance through the Emerging Infections Program (EIP) and receives isolates of GAS from hospitals, laboratories, and local health departments. WC also receives isolates from healthcare-associated investigations. In the past 6 years, over 2,200 GAS isolates have been received, including isolates from over 100 outbreaks.

**Methods:**

These isolates are analyzed using multiple methods for identification and characterization, including antimicrobial resistance (AR) and whole genome sequencing (WGS). Antimicrobial susceptibility testing (AST) is performed using the bioMérieux E-test gradient strip to assess increased resistance to CLI and ERY.

**Results:**

To date, WC has tested over 1,400 GAS isolates and identified CLI resistance in 130 isolates and ERY resistance in 230 isolates. Inducible clindamycin resistance (ICR) in GAS can develop when the pathogen is exposed to other antibiotics, such as ERY, and can result in treatment failure. To address this concern, the ICR test is currently under validation and will be used alongside the E-test when applicable. In addition to culture AST, isolates are sent for WGS and analyzed using an AR bioinformatic gene detection pipeline to screen for genes potentially leading to AR in strains circulating in New York State (NYS). To date, this method has identified over 400 samples containing one or more AR genes. Data analysis is underway to assess the relationship between the presence of AR genes and the correlation with phenotypic AST results.

**Conclusion:**

The surveillance testing performed at WC represents an extensive testing algorithm that can be used for the identification of trends in antimicrobial resistance in GAS. Data analysis may lead to the development of new assays to improve testing algorithms at WC and other public health laboratories.

**Disclosures:**

All Authors: No reported disclosures

